# Findings from Diet Comparison Difficult to Interpret in the Absence of Adherence Assessment. Comment on Tricò et al. Effects of Low-Carbohydrate versus Mediterranean Diets on Weight Loss, Glucose Metabolism, Insulin Kinetics and β-Cell Function in Morbidly Obese Individuals. *Nutrients* 2021, *13*, 1345

**DOI:** 10.3390/nu13113694

**Published:** 2021-10-21

**Authors:** Matthew J. Landry, Anthony Crimarco, Christopher D. Gardner

**Affiliations:** Stanford Prevention Research Center, Department of Medicine, School of Medicine, Stanford University, Stanford, CA 94305, USA; matthewlandry@stanford.edu (M.J.L.); crimarco@stanford.edu (A.C.)

We read, with interest, the recent publication by Tricò et al. on the effectiveness of two calorie-restricted eating patterns on weight loss and glucose homeostasis in individuals with morbid obesity and insulin-resistance [1]. The authors address a timely topic. However, we feel there are important limitations worth noting that impact the validity of the study’s overall findings.

When interpreting studies comparing different dietary patterns, adherence to assigned diets is a critical factor to consider [2]. As designed, the parallel arms in this study compared a Mediterranean diet (55% carbohydrate, 15% protein, and 30% fat) to a low-carbohydrate diet (30% carbohydrate, 30% protein, and 40% fat), with calorie restriction tailored to be 50% of estimated resting energy expenditure [1]. Adherence to study diets was determined to be adequate given that “actual weight loss was close to the predicted”. This is not an appropriate approach to assessment of adherence.

As we have recently discussed, it is common for participants to have difficulty achieving prescribed research diets in a free-living setting, which makes reporting the actual dietary intake and assessing adherence critical for interpretation [2]. Greater dietary adherence, regardless of the type of diet, is an important factor in weight-loss success and maintenance [3,4,5]. We recently conducted an intervention trial comparing a low-carbohydrate (ketogenic) and a Mediterranean diet in which we published a separate methods and adherence paper [6]. Even when we were providing participants with a food delivery service for 4 weeks to maximize adherence to their research diets we observed a wide range of adherence variability among participants; overall adherence was shown to be similar, increasing internal validity.

It is notable that the design of the study diets likely involved an inherent difference in engagement or effort to achieve adherence. Although baseline diet of participants was not reported, it appears likely the low-carbohydrate diet arm involved more extensive changes from usual diet relative to the Mediterranean diet arm. This raises the concern that differences in study outcomes between the two diet groups could be attributable to more than just the nutrient composition—differential engagement might explain some of the between diet differences [2,5].

Lastly, an additional key aspect for interpretation of broader generalizability is an assessment of the relative satisfaction of participants with their assigned diets. We feel it’s a missed opportunity for Tricò et al. to not report on the satisfaction participants have with study diets or on other potentially relevant individual-level factors (e.g., cost, familiarity). The practical implications for motivating food factors and values within interventions has been recently examined by Eustis and colleagues [7]. In our own trials, we have found varying psychosocial, demographic, and environmental motivational factors regarding adherence to study diets [6,8,9].

We hope that these comments stimulate a greater discussion by interventionists of strategies and assessments related to dietary adherence, and that they may want to incorporate these comments into future nutrition intervention studies.

## Data Availability

Not applicable.
